# Religion, politics and COVID-19 risk perception among urban residents in Malawi

**DOI:** 10.1186/s12889-022-13858-7

**Published:** 2022-07-27

**Authors:** Emmanuel Chilanga, Mastano Dzimbiri, Patrick Mwanjawala, Amanda Keller, Ruth Agather Mbeya

**Affiliations:** 1 Faculty of Social Work, University of Calgary, Edmonton, Canada; 2 College of Education, Health, and Society, Miami University, Oxford, USA; 3 Department of History, Miami University, Oxford, OH USA; 4 School of Social Work, McGill University, Montreal, Canada; 5 Department of Basic Science, University of Livingstonia, Livingstonia, Malawi

**Keywords:** Risk perception, Religious and political beliefs, COVID-19, Urban Malawi

## Abstract

**Introduction:**

Majority of Malawians have not yet adopted COVID-19 mitigation measures despite having knowledge about its infectivity, morbidity, and fatality. Understanding drivers of hesitancy to adoption of COVID-19 mitigation measures is critical as it can inform prevention programs. This study explores Malawians’ COVID-19 risk perception, and the associated constraints in the adoption of mitigation efforts. A Health Belief Model (HBM) approach was used to understand perceived factors that undermine public health COVID-19 messages to reduce the spread of the pandemic in Malawi.

**Methods:**

The study applied rapid appraisal and photovoice qualitative inquiry to comprehend risk perception regarding COVID-19. We purposively selected 52 participants from three major cities in Malawi. Audio and video interviews were transcribed verbatim, and transcripts were coded manually to derive key themes and concepts.

**Results:**

The study identified that social factors particularly religious and political beliefs influenced COVID-19 risk perception. Specific religious beliefs pertaining to individuals recognizing signs of the ‘Christian apocalypse’ were particularly associated with lower risk perceptions. Politically, participants believed COVID-19 lockdown measures were a ploy by the then-ruling party to remain in power.

**Conclusion:**

The study suggests that religious beliefs and political environment undermine self -perceived risk of contracting COVID-19 among urban dwellers in Malawi. We recommend that diverse actors in Malawi should collaborate to promote the dissemination of accurate COVID-19 discourses and reduce the severity of the pandemic’s impact in Malawi.

## Introduction

The coronavirus pandemic (COVID-19) is affecting health, social and economic welfare around the world. Economically developing countries will face unique challenges during the pandemic, particularly if they are unable to effectively slow the spread of the illness [1]. Malawi documented and registered cases of the COVID-19 later than most other African nations. As of 24 June 2022, a total of 86,204 people were tested positive and 2644 died from the disease [2]. Although the country reported no cases by March 20, 2020, Peter Mutharika as a President of Malawi during that time declared the state of national disaster on this date. By April 2, 2020, Malawi registered 3 confirmed cases of the coronavirus while the state of disaster remained in effect [3]. The Malawi government applied broad measures to mitigate its spread. These measures include declaration of a national state of disaster, instituting a COVID-19 ministerial task force, indefinite closure of educational institutions, and minimizing the number of public gatherings to a maximum of 100 people. The government also declared a full lockdown [4]. In reaction to the impending lockdown, many Malawians took to the streets to protest the president’s directives. Consequently, a grouping of human rights defenders obtained a court injunction preventing the government from enforcing a lockdown prior to addressing the basic needs of the people [5].

COVID-19 disease develops following exposure to SARS-CoV-2 virus and was first diagnosed in Wuhan city, China in December 2019 [6]. Following which WHO declared the COVID-19 outbreak a public health emergency of international concern on 30th January 2020 [7]. WHO labelled the COVID-19 outbreak, a pandemic on 11th March 2020 and as of 03 July 2020, over 10, 845, 275 cases of COVID-19 have been reported with about 521 113 deaths [7]. To avert COVID-19 epidemic related catastrophes, health experts on COVID-19 pandemic convened in Geneva on 11 to 12 February 2020 to identify key knowledge gaps. Consequently, they identified eight areas of study which broadly focus on diagnosis, prevention, treatment, and communication strategies [8].

WHO’s research and development (R&D) Blueprint, aims to facilitate the interactions between health experts and scientists to accelerate the dissemination of scientific knowledge and the social sciences must play a critical role to understand the sociocultural factors influence the pandemic [9]. Globally, there are emerging COVID-19 social concerns undermining disease prevention strategies. Low risk perception is one social factor identified as undermining adherence to COVID-19 mitigation measures in Malawi and globally [5, 10]. In America, factors such as peer pressure, perceived severity of disease outcome and conflicting messages are some of the predisposing factors for low-risk perception of COVID-19 contraction among the youth [11], In China, a study by Qian and Li [12] found that participants that had elevated levels of COVID-19 anxiety and fear have higher risk perception of contracting the disease. Other studies have revealed that an increase in public government mistrust decreases the risk of contracting COVID-19 [13]. We carried out this study to improve the understanding of social and political factors that undermine the adoption of COVID-19 mitigation measures, by focusing on risk perception of contracting the disease among urban residents in Malawi. Thus, the study aimed at answering the following research questions: 1) How do people perceive COVID-19 and the risk of getting infected, and 2). What are the reasons underlying the level of perceived risk towards COVID-19?

### Conceptual framework

A Health Belief Model (HBM) is used to understand the attitude of urban Malawians concerning their perception about the extent of how they feel at risk of contracting COVID-19 [14]. The HBM, developed in the early 1950s, has been used to describe change and sustenance of health-related behaviors. The model is used to guide health related behavior interventions considering the following aspects: (1) susceptibility to illness as perceived by an individual, (2) its severity (3), perceived behavioral change benefits, and (4) perceived barriers towards taking an action (Green & Murphy, 2014). Thus, the central idea of this model is that individuals make their own self-assessment regarding the risks and benefits associated with behavioral change once provided with information and recommendations given by authorities or experts. The HBM clarifies the reasons people engage with disease prevention strategies [14].

We focused our inquiry on the susceptibility to illness, perceived behavioral change, and associated barriers with little attention to its severity. Thus, at the time of conducting this study, COVID-19 was still in its early stages in Malawi, consequently people questioned the risk of infection. We explored whether Malawians felt at risk of contracting COVID-19 and explored their feelings about the disease. We explored participants’ barriers to adopt COVID-19 prevention strategies and participants’ access and trust in media and government campaigns. Furthermore, we assessed self-efficacy by examining participants' ability to overcome barriers inhibiting COVID-19 mitigate efforts. Recently, the HBM tool measured COVID-19 pandemic related mental health needs in Pakistan [15] and our study applies this tool in Malawi.

Risk perception refers to the subjective judgement and its underlying reaction against a specific threat. It represents threats and uncertainties that arise because of some changes in the health, social, or the political nature of the society [16]. Any piece of information that updates people about incoming diseases, such as COVID-19, triggers a perceived risk analysis. People’s perception of their individual risk can help individuals decide about mitigation efforts [17]. COVID-19 risk perception varies between countries, cultures, and individuals because of several factors. A study conducted in ten countries shows the highest levels of perceived risk to COVID-19 among people in the UK compared with other economically developed countries such as the USA, citing trust in government as one of the influencing factors [18]. An evaluation of the perceived risk to COVID-19 among college students in China revealed that specific groups of students had higher risk perceptions, such as females, those with knowledge of the disease, and students from Hubei [19]. Hedima et al. [20] found that risk perception in Nigeria is higher among females, health care workers, and young adults. However, a study conducted in Ethiopia using a Health Belief Model revealed that over 66.6% of the participants perceived COVID-19 as a low threat to their well-being [21]. Meanwhile, it is worthy to note that high-risk perception results in a more proactive response, and lower risk perception reduces the probability of taking preventative actions [22]. Accordingly, understanding COVID-19 risk perception is a critical piece of information, as it can help inform preventative measures.

### Political situation in Malawi related to COVID-19

**Fig. 1:**
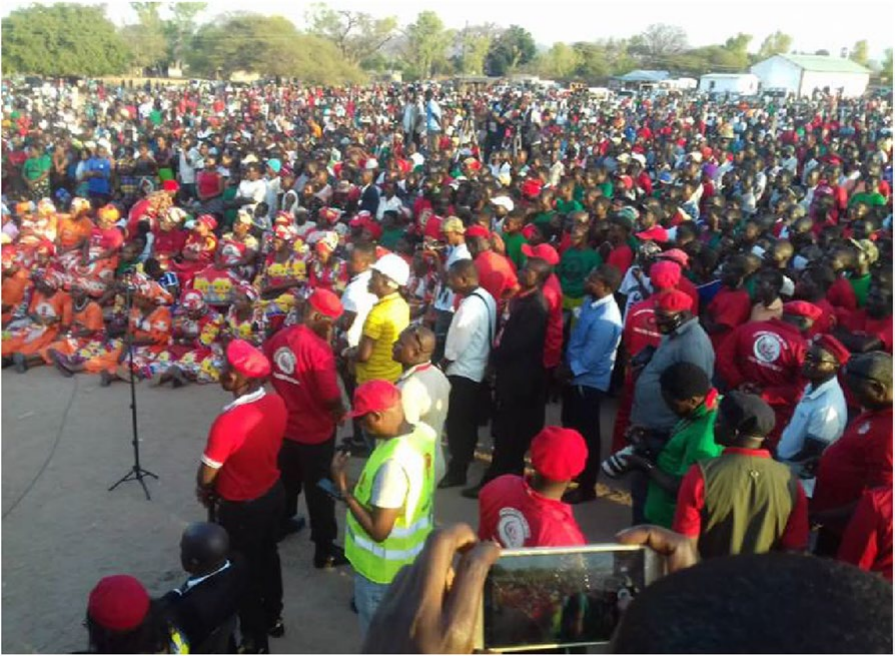
Tonse alliance political rally in Lilongwe city, Malawi, amidst COVID-19 epidemic. *Photo credit:* research participant, June 20, 2020

Malawi has witnessed political unrest since the 2019 general elections. Citizens protested the chairperson of Malawi Electoral Commission, Dr. Justice Jane Ansah, who pronounced Peter Mutharika as the president-elect. The Human Rights Defenders Coalition, an organization that champions human rights, spearheaded the Anti-Jane Ansah Movement. This organization encouraged millions of Malawians to protest in the streets. As the number of demonstrations swelled, leaders of the opposition appealed to the high court in demand for justice. On February 3, 2020, the high court nullified the presidential outcome of the May 2019 general elections and granted a 150-day period for fresh elections [23] in which Dr. Lazarus Chakwera, the opposition leader, was formally declared a winner. The change in power took place on 27th June 2020. It is during this period of political unrest that COVID-19 emerged in Malawi [24]. Politicians facing an ethical dilemma made a judgement call on whether to conduct public political rallies. Politicians opted for massive open public rallies, disregarding COVID-19 mitigation measures (Fig. 1), and this suggests that they had either a lower risk perception or that politicians prioritized their political interests over public health concerns [22].

Socio-cultural factors and the political challenges faced by the Malawi government to reinforce COVID-19 mitigation measures resulted in a surge of the cases of the disease in June, as the local transmission surpassed imported cases. On Tuesday, 12th January 2021, the Malawi government declared a state of national disaster, and this announcement came after the deaths of two senior Cabinet ministers and many top government officials related to COVID-19 [25]. There is a marked probability that the COVID-19 cases in Malawi are under-represented and this could be due to poor health systems and infrastructures. The government continues to warn its citizens that they need to regard everyone as the carrier of the SARS-CoV-2.

**Fig. 2:**
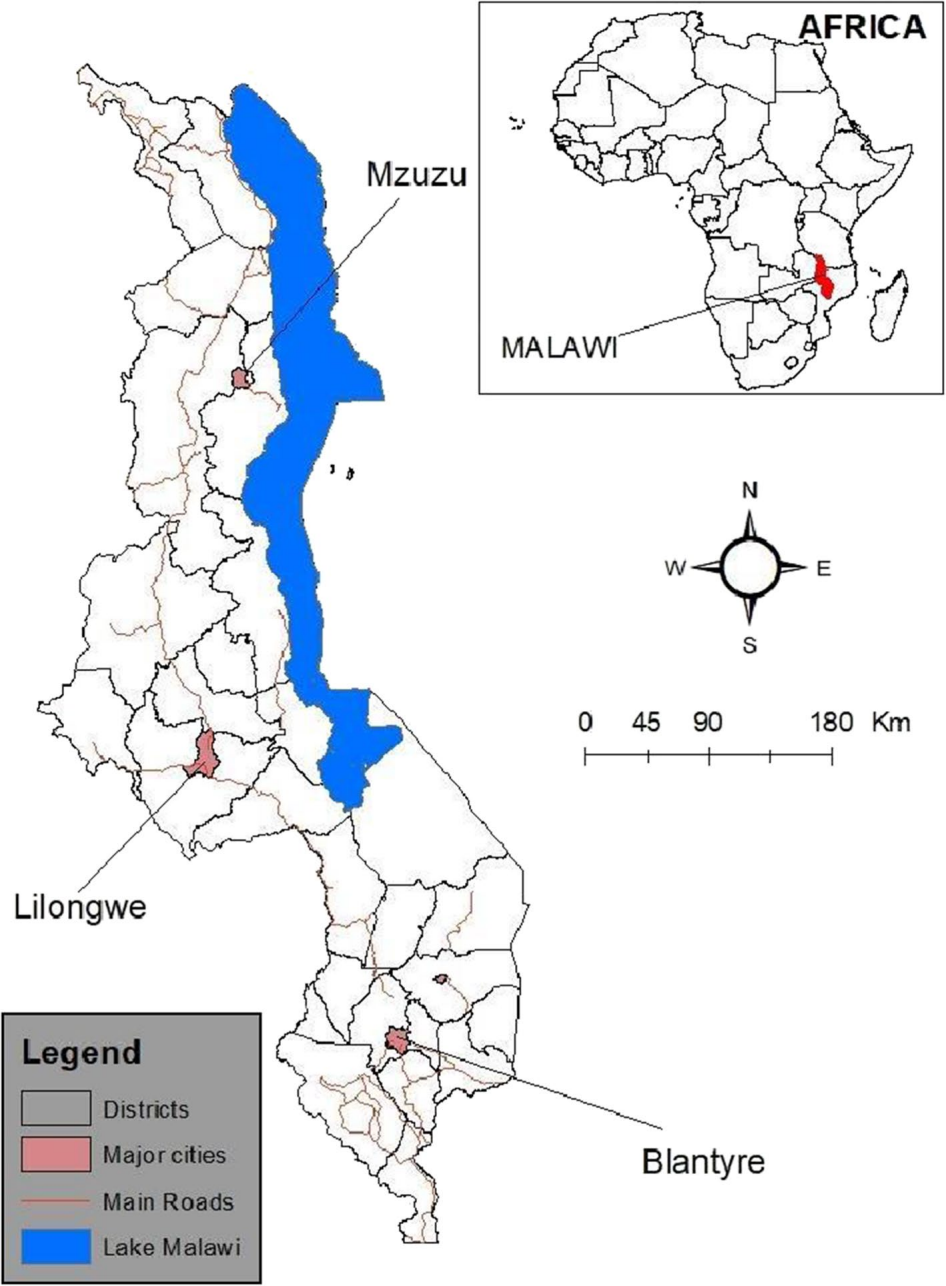
Location of study cities in Malawi

All the 28 administrative districts of Malawi have had cases of COVID-19, the three major cities of Blantyre, Lilongwe and Mzuzu (Fig. 2) are the epicenters of the disease [5]. Understanding community adherence to COVID-19 preventive measures is critical, as Malawi health facilities cannot manage an influx of critical cases because of the limited health infrastructure [3].

## Methods

The study applied a rapid appraisal research approach [26] to develop a qualitative understanding of the barriers to COVID-19 mitigation measures in Malawi. This method uses many of the characteristics of qualitative research. Thus, it incorporates two or more multidisciplinary researchers; team interaction is an aspect of the method, and as a result, it produces faster and more comprehensive outcomes [27]. Rapid appraisal offers a systemic perspective used to understand factors that undermine participants’ adoption of COVID-19 mitigation recommendations. This approach also allows triangulation of multiple data sources [28]. The approach also allowed us to have an iterative data collection and analysis process, as the COVID-19 management procedures were evolving in Malawi. Therefore, the approach provided us with a flexible but rigorous approach to the collection and analysis of our qualitative research data related to COVID-19 risk perception among urban dwellers in Malawi.

### Study location

We conducted the study in three urban cities of Malawi, namely Mzuzu, Lilongwe and Blantyre, where cases of COVID-19 are high [3]. Malawi is situated in the southern part of Africa, bordered by Tanzania to the north, Mozambique to the southeast, and Zambia to the northwest (Fig. 2). Estimates show that Malawi has a total population of 19,737,191as of September 2021 [29]. Poverty is widespread across the country as more than half of the population lives below the poverty line (less than $1.90) and about a quarter are ultra-poor households [29]. Majority of the population work in the agricultural and informal sectors, which form the backbone of Malawi's economy besides foreign aid.

### Data collection and analysis (Created by Authors in ArcMap, 2020)

We collected data from May to June 2020 [30]. We employed convenience sampling technique and selected the research participants based on the fundamentals of maximum variation [31] to document diverse variations relating to the risk perception of contracting COVID-19. The purposive selected demographic attributes included age, marital status, education, professional, city, religion, and political affiliation and was informed by our prior study in Malawi [32]. Fifty-two participants (*n* = 52) were engaged in the interview process drawn from Mzuzu, Lilongwe, and Blantyre cities of Malawi. Local community organizations on the ground helped to identify the participants [30, 33–35] and we advertised our study on WhatsApp groups. To mitigate the transmission of COVID-19 during the research study and adhere to the lockdown regulations put in place by the Malawi government, all interviews were conducted and recorded with WhatsApp mobile platform. WhatsApp technology has become an important platform in developing countries where internet connectivity is a challenge [36]. Participants signed the informed consent prior to participation of the study.

An adapted standard risk perception of an infectious disease outbreak interview guide was used to collect data [37]. The questions were modified to capture the key constructs of the Health Belief Model. To ensure validity and reliability of our instruments, we first sent the interview guides to some people and experts for pre-testing. After we received the responses, the research team analyzed and compared the results. Where there were minor discrepancies in the vocabulary, we consulted other language and health experts who helped us to address the existing inconsistencies. After revising the questions as advised by the experts, we then sent it for actual data collection.

The questions explored the views of the respondents about their knowledge of COVID-19, and if they perceive themselves to be at risk of contracting the infection. Interviewers also asked respondents about their willingness to adopt COVID-19 preventive efforts, such as hand washing, wearing of face masks, and physical distancing. First, we transcribed the interviews verbatim into Chichewa and Chitumbuka local languages. Subsequently, we translated the transcription into English. To ensure continued immersion and rigor in our qualitative data we analyzed the transcripts using hand-coding. First, each author read and reread the raw data line-by-line and then derived codes relevant to our research questions [38]. We compared the codes for consistency, and later we organized and linked emergent codes into four broader themes. For data triangulation, photos and videos were used to elicit participants’ imagination on how they perceived themselves at risk of contracting COVID-19. This helps us to have multisensory experience of participants’ narratives. We performed member checking with our local research team and incorporated gatekeepers’ insights regarding predisposing factors of low-risk perception of local people in Malawi [39].

### Research Ethics Review

The University of Livingstonia research ethics committee approved this research project (protocol number: UNILIA-REC/1/CUP 2/01[40]. We sought written permission from all three city assemblies. Informed written and oral consent was obtained from all the participants prior to the interview.

## Results

At the time of the interview, participants ranged in age from 21 to 64 years. A total of 27 women and 25 men were included in the study. Table 1 provides sociodemographic characteristics of the study participants.

**Table 1:** Social-demographic characteristics of study participants (*n* = 52)

**Variables**		***N* = (52)**	**%**
Gender	Women/Female	27	52
	Men/Male	25	48
Age	18–30	17	33
	31–60	28	54
	60 +	7	13
Marital status			
	Single	18	35
	Married	34	65
Education			
	Primary	13	25
	Secondary	23	48
	Tertiary	16	31
Professional			
	Business	31	60
	Academics	4	8
	Health related field	17	33
City			
	Blantyre	17	33
	Lilongwe	17	33
	Mzuzu	17	33
Religion			
	Christian	31	60
	Moslem	14	27
	Others	7	13
Political affiliation			
	DPP alliance	21	40
	MCP alliance	31	60

The results of our study include quotations, to illustrate how participants attached meaning to each emerging theme. We selected the quotations using the criteria suggested by [41] in Malawian context that include: (1) the ability to represent divergent perspectives; (2) typical views expressed by many respondents; and (3) the depth or clarity with which the ideas are conveyed. Pseudonyms have been used to protect confidentiality of the research participants. Our study identified four overarching themes that appear to undermine participants’ risk perception of COVID-19 epidemic. In this paper, we employed the health belief model to understand people’s perception of risk and their action to prevent the disease.

Our results revealed that several beliefs influenced perception of COVID-19 risks among the research participants. The following paragraphs present the common themes that are related to low and high-risk perception of COVID-19 among urban dwellers in Malawi.

### Religious beliefs

Our study releveled a number of risk factors related to religious beliefs that influenced risk perception of COVID-19 in urban Malawi. One of the low risk factors was linked to the belief of coming of Christ. Some Christian participants understand that Jesus, the son of God, warned the world about his return to judge humanity and one signal of his return is a global pandemic. Therefore, some respondents ascribe to this prophecy and maintain that COVID-19 is fulfilling the Christian Holy Scriptures marking the end of days. The following excerpt from a pastor in Mzuzu city illustrates this view.

“This is not a real virus like HIV; rather, it is a disease from the darkness. The evil spirits have brought this disease to attack only those who are not righteous. This is a sign of the coming of Jesus to redeem righteous people in the world and the police are accomplices of this prophecy” [Leah, Businesswoman, 47 years, Mzuzu].

Consistent across faiths, multiple participants reporting those who do not pray and have little or no faith in God are more likely to be infected by COVID-19, unlike believers. One local leader reported, the only way to avoid COVID-19 is to repent, which is a perception in sharp contrast to scientific preventative measures. Some participants ascribed to the theory that COVID-19 has emanated from the wrath of God. They claimed that the sins of the world have angered God. Furthermore, most of the respondents reported that God wants to wipe out humankind from the earth through the epidemic. They substantiated their line of narrative regarding the biblical stories of Noah and the fate of Sodom and Gomorrah. In all these given scenarios, they claimed God used heavy rain and fire to destroy the sinful people. These religious participants perceived that COVID-19 is an act of God. One key respondent from Islamic faith explained this view while showing a photo of destruction of Sodom and Gomorrah:

“This pandemic is Kiamah (punishment) from Allah to humankind for their sins. Allah has been tolerating the sins that people have been committing, such as stealing of government funds, prostitution, and murdering of albinos in this country. God has seen that people are not repenting, and he wants to punish them” [Chejuma, 36, businessman, Blantyre].

Religious ideologies undermined perceived risk of contracting COVID-19 among the participants. According to half the participants, the onset of COVID-19 fulfills the biblical prophecy in the book of Revelations. One participant shared, the book outlines in the days prior to the return of Jesus, people will experience many horrible things. One further potential sign is the number 666, which is considered the number of the beast. They purport that “antichrist people” will force this number upon Christians. The accompanying excerpt illustrates a purported connection between 666 and COVID-19 pandemic.

“The government last year told us we should get the national identity cards so that everyone should have a number. They said that without that number we cannot access social services. This year they say there is COVID-19, which is claiming the lives of people. To avoid it, the government has shut down churches. This is strange, as God commands us to pray all the time. This government wants us to stop praying; perhaps the numbers on the national cards will work now. I cannot follow their advice, as I do not want to get the 666 number” [Chisomo, 21, businesswoman, Lilongwe].

Some religious participants voiced their doubts about COVID-19 vaccination research. Sharing that God’s followers should not take part in the trials. One participant suggested that the COVID-19 vaccine would induce a mark of triple six, a negative omen among devout Christians. Another respondent assumed that COVID-19 is a disguised Roman numeral number 666 that has come through this epidemic. He suggested that antichrists have mixed the roman numerals C = 100, VI = 6, D = 500, and minus 19 to prevent people from understanding that it is the number 666. Therefore, religious beliefs influence some participants and ultimately counter public health information and initiatives. Although participants showed knowledge of risk factors and mitigation methods, they did not consider themselves at risk because of religious beliefs and political affiliations. Many reported that, if they remained devout, they could not contract the disease.

### Political beliefs

Two polarizing views emerged from Malawians based on their political affiliation concerning their risk perception associated with contracting COVID-19. Respondents’ views were linked to the principle of cost and benefits within the broader HBM framework. Our findings shows that a high-risk perception of COVID-19 was seen as beneficial to the then-ruling party, as its leadership would remain in power if they cancelled the presidential elections in June 2020. However, compliance with isolation strategies was perceived to undermine the capacity of opposition parties to challenge ruling party through the democratic election. Participants who supported the then-ruling party claimed that COVID-19 is real and the prevention measures that the government planned were in the best interest of Malawians. In this example, the participant held the view that they were at high risk of contracting the disease and justified lockdown measures. The following scenario exemplifies this line of reasoning as a participant showed the pictures of opposition party political rallies.

“The opposition parties are reducing concerted government efforts towards combating COVID-19. If you can see, they challenged the presidential order of lockdown and they have started their public political campaigns. Our president is leading by example as he is on self-quarantine. Our presidential running mate is distributing facemasks whenever he is conducting political rallies” [Christopher, 25, Businessman, Blantyre].

We observed that the participants that self-identified with political opposition parties claimed that there were no COVID-19 cases in Malawi as they argued that the then-government wanted to capitalize on the crisis. Yet, the issue that the illness is fake or exaggerated created genuine concern for some of our participants. In Malawi, there have been no public declarations of COVID-19 infections. Lack of COVID-19 disclosure undermines the perceived risk of contracting the disease by some participants. The following excerpt illustrates this point of view as supported by a newspaper photograph.

“There is no COVID-19 in Malawi. Look in Britain, the Prime Minister Boris Johnson disclosed that he was suffering from coronavirus. We could see on television how his condition was deteriorating. What is so special with Malawi that even a single person has come to the open to say he or she is suffering from it? This is just government propaganda to shun away from the presidential election” [Phiri, Businessman, 26, Mzuzu].

The excerpt suggests that some opposition political leaders encouraged people to patronize their political rallies. Six participants added that top official of the then-coalition opposition parties encouraged political patrons to hug each other as a proof that there is no COVID-19.

This approach undermined risk perception among supporters of the opposition parties. The interviews with health professionals show a higher risk perception attributed to COVID-19 regardless of their political party affiliation. The following excerpt illustrates the risk perception in one of the health care workers:

“We had a COVID-19 orientation, and we were told that the disease is dangerous and deadly, hence the need to observe and follow all the precaution measures. For example, we need to practice social distancing -thus sitting 3 meters apart from each other, and no handshakes. People should always put masks on their face, and they should frequently wash their hands”. [Neri, 31, Healthcare, Lilongwe].

Neri’s statement suggests that some health professionals in Malawi accepted public health recommendations and ascribed to COVID-19 preventative measures.

The final theme that emerged is lack of trust in the government. Rampant corruption reduced public trust in the government in Malawi, which undermined citizens’ perceived risk of contracting COVID-19. Some participants claimed that some donor organizations remitted COVID-19 funds to countries that registered the disease in Africa. Reporting that, in Malawi, there was no case of the epidemic before April 2, 2020, and that the government colluded with health workers to claim that some people were tested positive for COVID-19. These participants felt politicians would use COVID-19 funds for self-enrichment. One participant recounted a record of two government Ministers caught on camera on national television discussing ways of concealing evidence that they are collecting COVID-19 allowances.

“My suspicion that this government fabricated that there is COVID-19 in Malawi for them to get rich out of this pandemic was verified yesterday on MBC TV. The minister of health, Jappie Mhango and information minister, Mark Botomani were not aware that microphones were turned on during COVID-19 briefing session. Believing they were speaking confidentially, Botomani warned Mhango that their tactic of withdrawing funds is about to be revealed to the public. I encourage you to access this link to understand more. https://www.nyasatimes.com/hrdc-gives-cabinet-ministers-botomani-and-mhango-7- days-to-resign/” [Thandizo, businessman, 34, Blantyre].

Thandizo’s sentiments about the behavior of the two ministers undermined her risk perception of contracting the disease. The two ministers were discussing how they can manipulate the COVID-19 funds as part of their allowances. Yet, the then-ruling party denied that ministers were not being getting the allowances drawn from the COVID-19 funds.

## Discussion

Our study shows that participants’ risk perception of contracting COVID-19 is affected by multiple social factors. Dominant religious and political beliefs are leading undermining factors while being a medical professional increases risk perception. Religious ideologies that most influenced risk reduction measures were the belief that this disease is a punishment from God, or a fulfillment of biblical narratives, and that healing, and prevention can come from being steadfast in prayers.

Political affiliation to an opposition alliance parties and distrust in the government seemed to lower self-perceived risk of contracting the disease. Using the constructs from a health belief model, we posit that people with strong religious faith do not see themselves at risk. Consequently, they resist taking preventive measures to mediate the spread of the disease. Our results also found that many religious leaders report they consider themselves immune to the infection. Malawi is deeply a religious country with most citizens affiliated with religious groups. As a result, we propose that officials must consider religious influences and integrate religious leaders in planning public health protocols and dispersing public health knowledge. This strategy was used in Bhutan and showed some success in promoting COVID-19 vaccination acceptance [42].

Our findings are consistent with a study by Lichtenstein et al. [43] who found that religious and political beliefs undermine COVID-19 management in many African countries, as well as in some affluent countries such as the USA [18]. For instance, Liechtenstein revealed that in Nigeria, an Islamic scholar by the name of Abubakar Imam Aliagan challenged the Nigerian government’s mitigation efforts specifically as it pertained to mosques and other religious institutions. He claimed Muslims are endowed with spiritual power to fight the virus. The same study referred to President John Magufuli of Tanzania, who proclaimed that COVID-19 is a pandemic from the devil. He recommended churches not only remain open, but that people attend to strengthen prayers and avert the epidemic.

Since our findings support prior studies that religious faith and politics play a critical role in shaping COVID-19 risk perception in Africa, there is an urgent need for policy makers to work collaboratively with religious and political leaders to understand their needs and concerns while simultaneously working to ensure they provide accurate information about the causes, prevention, and risk of the epidemic. We strongly support the ideas of Ogola [44] who claimed that many COVID-19 messages reaching the population are misinformation coming from several actors. In Malawi, religious leaders and politicians are spreading this misinformation. It is important to note that a study in Korea found that religious affiliation is not a precursor of COVID-19 immunity, as members of Shincheonji religious grouping were infected by the disease [45]. Cases of religious institution-based outbreaks may be helpful in dispelling the claim that COVID-19 is a manifestation of God’s anger and can be avoided by being a religious believer. This study also underscores the dilemma that politicians and health experts are encountering across the globe to balance the individual rights of citizens while also promoting health and wellness [46]. In Malawi, people reported failing to adopt COVID-19 preventive measures because of their religious and political affiliations. Furthermore, our findings demonstrate political party leaders mainly from the opposition side then contributed hugely to misinformation at times, claiming the absence of the coronavirus in Malawi as they encouraged people to attend large political rallies [5]. In the context of a political movement that coincided with the outbreak of the illness, this untimely event polarized the population, with many people choosing to express their political freedoms over following the health directives [5]. After the then-opposition party gained power, their message about COVID-19 changed, once elected they confirmed that the disease is real and encourage people to follow guidelines from the public health experts. We, therefore, posit that the current Malawi government should strive to build public trust and work with journalists against misinformation.

The government needs to work in collaboration with religious and community leaders to engage in culturally sensitive dialogue while working to minimize competing interpretations of the disease. Rosenthal et al. [47] proposed that African COVID-19 management programs must ensure community ownership, addressing its economic impacts, and maintaining health services for non-COVID-19. In Malawi, the politics of COVID-19 management partly contributed to the loss of presidential elections by the then-ruling party. Opposition parties took advantage of COVID-19 crisis and capitalized the narrative of corruption and mismanagement of government resources to their own benefit [33]. The capitalization of a public health crisis for political gain represents a serious health risk. While the misinformation spread by various religious actors in Malawi remains another important risk. We therefore suggest that the current government of Malawi rebuild the damaged government reputation by ensuring that COVID-19 management is holistic, and people centered. Malawians, like many Africans, will be critical of the government on how COVID-19 funds are managed, which will sway risk perception and mitigation compliance efforts [48]. The Malawi government should also work on building strategic partnerships with religious institutions to gain support to better disseminate information from health experts about COVID-19 to religious communities. Working with religious institutions may help to thwart further spread of the disease as observed elsewhere by Mohammadi et al. [49].

The study identified that regardless of political and religious affiliation, information from a health expert suggests that medical professionals in Malawi have a higher risk perception and are likewise knowledgeable about COVID-19 preventative measures. This finding is consistent with a study in Saudi Arabia where 72% of the health workers reported a high perceived risk of contracting the disease [50]. Therefore, COVID-19 risk perception among people in urban Malawi should be understood in the context of the existing socio-cultural factors such as religion and the political environment of between 2019 and 2020.

## Conclusion

Informed by a Health Belief Model as a conceptual framework, our study reveals that some segments of the population evaluate COVID-19 with a low-risk perception in three major cities of Malawi, reducing the probability of the effectiveness of the health measures at slowing the virus. Religious and political beliefs influenced participants towards a lower perception of risk. Political environment and corruption also inhibited the Malawi government’s ability to impose effective prevention methods. We suggest that the government develop partnerships with religious institutions to help spread right scientific approaches to the public. Furthermore, the current government needs to work towards reducing corruption in all government institutions, including increasing transparency with the management of COVID-19 funds to rebuild public trust. Reducing corruption and improving cross collaboration between religious institutions and medical experts would help to increase public awareness about the genuine risks of health crises such as COVID-19. These strategies would help to increase the population’s collaboration with COVID-19 mitigation efforts. Finally, there is a need to further explore the implications of risk perception for COVID-19 vaccine uses in Malawi.

## Data Availability

This study focused on a COVID-19 risk perception among urban dwellers in Malawi. For data accessibility, contact the corresponding author.

## Abbreviations

COVID-19: Coronavirus disease 2019
WHO: World Health Organization
